# Overexpression of Ginkbilobin-2 homologous domain gene improves tolerance to *Phytophthora cinnamomi* in somatic embryos of *Quercus suber*

**DOI:** 10.1038/s41598-024-70272-2

**Published:** 2024-08-21

**Authors:** Susana Serrazina, MªTeresa Martínez, Serine Soudani, Gonçalo Candeias, Marta Berrocal-Lobo, Pablo Piñeiro, Rui Malhó, Rita Lourenço Costa, Elena Corredoira

**Affiliations:** 1 Faculdade de Ciências, BioISI-Biosystems & Integrative Sciences Institute, Universidade de Lisboa, Lisbon, Portugal; 2 Misión Biológica de Galicia, Sede Santiago de Compostela, Consejo Superior de Investigaciones Científicas (MBG-CSIC), Avda Vigo S/N, 15705 Santiago de Compostela, La Coruña Spain; 3 Centro Para La Biodiversidad y Desarrollo Sostenible (CBDS), ETSIMFMN, Universidad Politécnica de Madrid, Ciudad Universitaria S/N, 28040 Madrid, Spain; 4 Instituto Nacional de Investigação Agrária E Veterinária I.P., Oeiras, Portugal

**Keywords:** *Agrobacterium tumefaciens*, Cork oak, Cysteine-rich repeat secretory protein, Genetic transformation, In vitro tolerance assays, Oak decline disease, Biological techniques, Biotechnology, Plant sciences

## Abstract

In recent decades an extensive mortality and decline of *Quercus suber* populations mainly caused by *Phytophthora cinnamomi* has been observed. In the current study, a chestnut gene homologous to *ginkbilobin-2* (Cast_*Gnk2-like*), which in *Ginkgo biloba* codifies an antifungal protein, was transferred into cork oak somatic embryos of three different embryogenic lines by *Agrobacterium* mediated transformation. The transformation efficiency varied on the genotype from 2.5 to 9.2%, and a total of 22 independent transformed lines were obtained. The presence of *Cast_Gnk2-like* gene in transgenic embryos was verified in all lines by PCR. The number of transgene copies was estimated by qPCR in embryogenic lines with high proliferation ability and it varied between 1 and 5. In addition, the expression levels of *Cast_Gnk2-like* gene were determined in the embryogenic lines, with higher levels in lines derived from the genotype ALM6-WT. Transgenic plants were obtained from all transgenic lines and evaluated after cold storage of the somatic embryos for 2 months and subsequent transfer to germination medium. In vitro tolerance tests made under controlled conditions and following zoospore treatment showed that plants overexpressing *Cast_Gnk2-like* gene improved tolerance against Pc when compared to wild type ones.

## Introduction

Cork oak (*Quercus suber* L.) is a very familiar evergreen oak from the western Mediterranean, where it occupies an area estimated to be over 2.1 million hectares^1^. Portugal, Spain, Morocco and Algeria together host more than 90% of the species' distribution area^2^. Cork oak trees can appear forming forests called Sobreirais in Portuguese and Alcornocales in Spanish or forming part of the agroforestry ecosystems called Montados in Portuguese and Dehesas in Spanish. Cork oak forests and dehesas constitute ecologically and economically sustainable systems as they serve as an important tool in desertification prevention and generate high levels of biodiversity^3^. From an economic point of view, cork oak is the only species used for industrial cork production^4^. Cork has been used by humans for more than 2,000 years^5^ and is the sixth most economically important non-woody forest material at the global level. More than 80% of the world cork is produced in the Iberian Peninsula^2,6^. Cork is lightweight, water-resistant, flexible, chemically stable and fire-resistant^7^. It is also renewable, recyclable and biodegradable^8^. The greatest application of cork is the production of stoppers for the wine industry and champagne, but cork is also used as a construction material, for floors and thermal insulation^2,6^. Its applications are so broad that even NASA uses it for the insulation of spaceships, and surfboards and skateboards are also made from cork^9^.

However, since the early 90’s cork oaks have been seriously affected by the oak decline dieback which seriously threatened their populations with a resulting decrease in cork production (losses ranged from 40 to 89%)^10,11^. Oak decline is a multifactorial disease in which many abiotic and biotic factors such as drought, floods, frost, over-grazing, low regeneration ability, insect pests, and pathogens interact in a scenario of global climate change^12^. Among them, the oomycete *Phytophthora cinnamomi* Rands (Pc) is considered the main cause^13^. It seems that environmental changes provoke physiological decline of the trees, and this decline significantly increases the tree vulnerability to Pc, insects and other pathogens^14^. To date, traditional agronomic practices have not been effective in controlling infection caused by this oomycete and the generation of tolerant genotypes seems the most suitable option. However, conventional improvement programs for forest species face an enormous limitation: the long generation time of forest species, which in the case of oak species can exceed 30 years, and the high level of heterozygosis^15,16^. In the short future, the European Parliamentary Research Service (EPRS) recommends the relaxation of the rules for the use of transgenic plants obtained by targeted mutagenesis, cisgenesis or intragenesis^17^. Consequently, genetic transformation with antifungal genes isolated in the same species or a closely related species could be a good alternative to conventional breeding efforts.

The efficient introduction of new characters in plants involves the development of an effective transformation protocol and an available regeneration method that permits the generation of a plant from the transformed cells^18^. In hardwood species, somatic embryogenesis (SE) is considered the best regeneration method for genetic engineering and cryopreservation procedures^19^. In the last two decades, considerable advances have been reported on the development of SE in cork oak, even from explants derived from adult trees^20,21^. In contrast, genetic transformation has been less studied, especially for the overexpression of genes of interest^15^. Until now, genetic transformation of cork oak has been achieved using somatic embryos from both mature and juvenile materials introducing marker genes^22,23^ as well as a gene to confer herbicide resistance^24^. However, resistance has only been tested in somatic embryos at laboratory level, as plantlet conversion was not reported. As far as we are aware, the only report of genetic transformation in cork oak with genes to confer resistance to Pc is the overexpression of *CsTL1,* which codifies a thaumatin-like protein, a pathogenesis-related protein^25^.

Since the specific genes involved in oomycete defense have not yet been identified in cork oak, it is necessary to look for alternatives to induce some type of resistance against Pc. There are many indications that the overexpression of genes that codify antifungal agents may confer tolerance to pathogen attacks^26–28^. Under the term antifungal agents are pathogenesis-related proteins, antimicrobial proteins and peptides, DUF26-containing proteins, and other proteins such as polygalacturonase-inhibiting proteins or albumins^29^. DUF26-containing proteins are characterized as containing cysteine-rich domains with a conserved C-X8-C-X2-C motif in which lies its ability to fight plant pathogens and especially fungi^29,30^. The most well-known DUF26-containing protein is ginkbilobin-2 (Gnk2) from *Ginkgo biloba* seeds, which is predictly secreted and inhibits the growth in vitro of *Fusarium oxysporum*, *Tricoderma reesei* and *Candida albicans*^31^. Gnk2 showed similarity to the extracellular domain of plant cysteine-rich receptor-like kinases, but not to pathogenesis-related proteins^32^. Its antifungal activity is attributed to the binding of the DUF26 domain to sugar residues in the fungal cell wall^33^. A candidate gene for Pc resistance, homologous to *Gnk2*, was identified in the root transcriptome of the resistant chestnut *Castanea crenata* Sieb. and Zucc.^34^. In *C. crenata*, *Cast_Gnk2-like* had a high expression in non-inoculated roots and was distinctly up-regulated 48 h after Pc inoculation, contrasting with the feeble expression in *C. sativa*^35^. Furthermore, the recombinant protein encoded by *Cast_Gnk2-like* delays Pc mycelial growth *in vitro*^36^. *Quercus ilex* L. plants overexpressing *Cast_Gnk2-like* gene were able to survive more days in the presence of Pc when compared to non-transformed controls^37^. The studies carried out with *Cast_Gnk2-like* are revealing an important role in the defense of susceptible *Fagaceae* to Pc. Here, we describe the development of transgenic cork oak somatic embryos and plantlets overexpressing the *Cast_Gnk2-like* gene through *Agrobacterium*-mediated transformation. We also evaluate the resistance of transgenic plants to inoculation with *P. cinnamomi*.

## Results

### Somatic embryo genetic transformation

**Figure 1:**
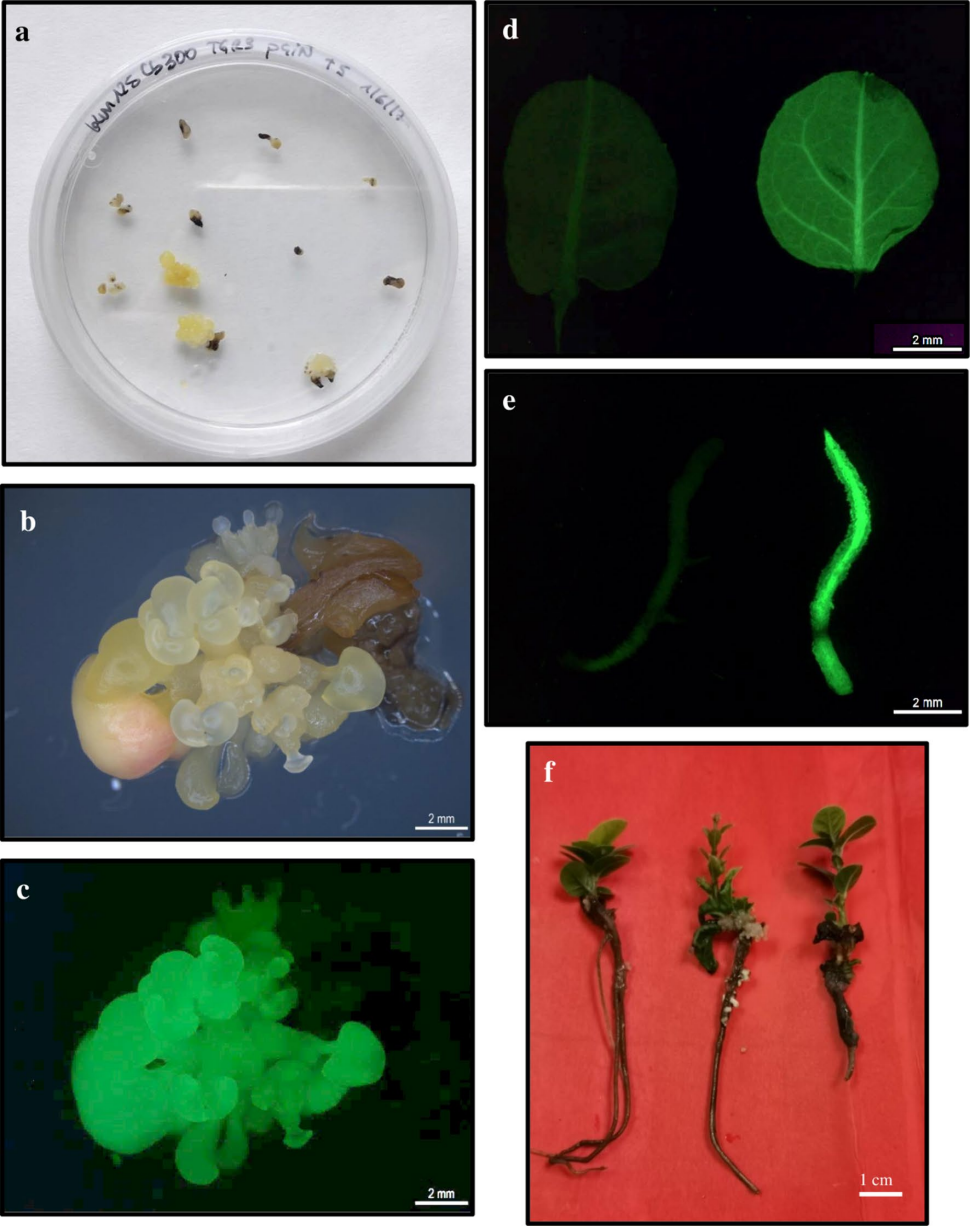
Genetic transformation of somatic embryos of cork oak. (**a**) Kanamycin resistant explants observed after ten weeks on selection medium. Diameter of the Petri dish, 90 mm. (**b**) Group of transgenic somatic embryos observed under white light. (**c**) The same group of transgenic somatic embryos observed under blue light showing green fluorescence. (**d**) GFP expression in leaves isolated from a non-transgenic plant (left) and a transgenic plant (right). (**e**) Expression of GFP in roots from a non-transgenic plant (left) and from a transgenic plant (right). (**f**) Transgenic plantlets (left and right) and non-transgenic plant (center) obtained from somatic embryos of genotype TGR3 following to two months of cold storage and eight weeks on germination medium. Bar, 1 cm.

Somatic embryos from three embryogenic lines were precultured for one day, cocultured for 5 days with the bacteria and cultivated on kanamycin-containing medium during 10 weeks. The first kan-resistant somatic embryos were observed after 4 weeks of culture on kanamycin-containing medium but only in line TGR3 (Fig. 1a). The first kan-resistant somatic embryos in lines ALM6 and ALM80 were detected later, after 8 weeks on selection medium. The best percentages of kan-resistant explants (14.2%) were also obtained with TGR3 line, although without significant differences with the values achieved with lines ALM80 (11.7%) and ALM6 (10%) (Table 1). The transformation efficiency (i.e. the explants GFP +) was also higher in line TGR3 (9.2%) than in lines ALM80 (6.7%) and ALM6 (2.5%) (Table1; Fig. 1b,c), but similar to the percentage of kan-resistant explants, no significant differences were observed between the three lines (Table 1). The negative controls (non-transformed explants but cultured on kanamycin-containing medium) died without forming callus or new somatic embryos, which indicates that the selection pressure is adequate.

**Table 1:** Effect of genotype on the percentage of kanamycin-resistant explants, on the transformation efficiency and the selection efficiency following culture in selection medium of cork oak somatic embryos transformed with the strain EHA105pKWG2D-GIN.

Embryogenic line	Kanamycin resistant explants (%)^1^	Transformation efficiency (%)^2^
TGR3	14.2 ± 2.8	9.2 ± 2.5
ALM80	11.7 ± 3.1	6.7 ± 2.7
ALM6	10.0 ± 3.5	2.5 ± 1.3
*ANOVA I*	ns	ns

Values are means ± SE of 12 Petri dishes with 10 explants each. ^1^Percentage of initial explants that are kanamycin-resistant after 10 weeks in selection medium; ^2^Percentage of initial explants that are GFP + after 14 weeks in selection medium.

ns: not significant.

After GFP evaluation, one somatic embryo at cotyledonary stage was isolated from each kan-resistant and GFP + explant. The selected embryos were proliferated independently on kanamycin-containing medium to establish the different transgenic lines. After, 2–3 subculture periods a total of 22 transgenic lines were obtained: 11 transgenic lines in TGR3, 8 transgenic lines in ALM80 and 3 transgenic lines in ALM6, that were maintained through secondary embryogenesis.

**Figure 2:**
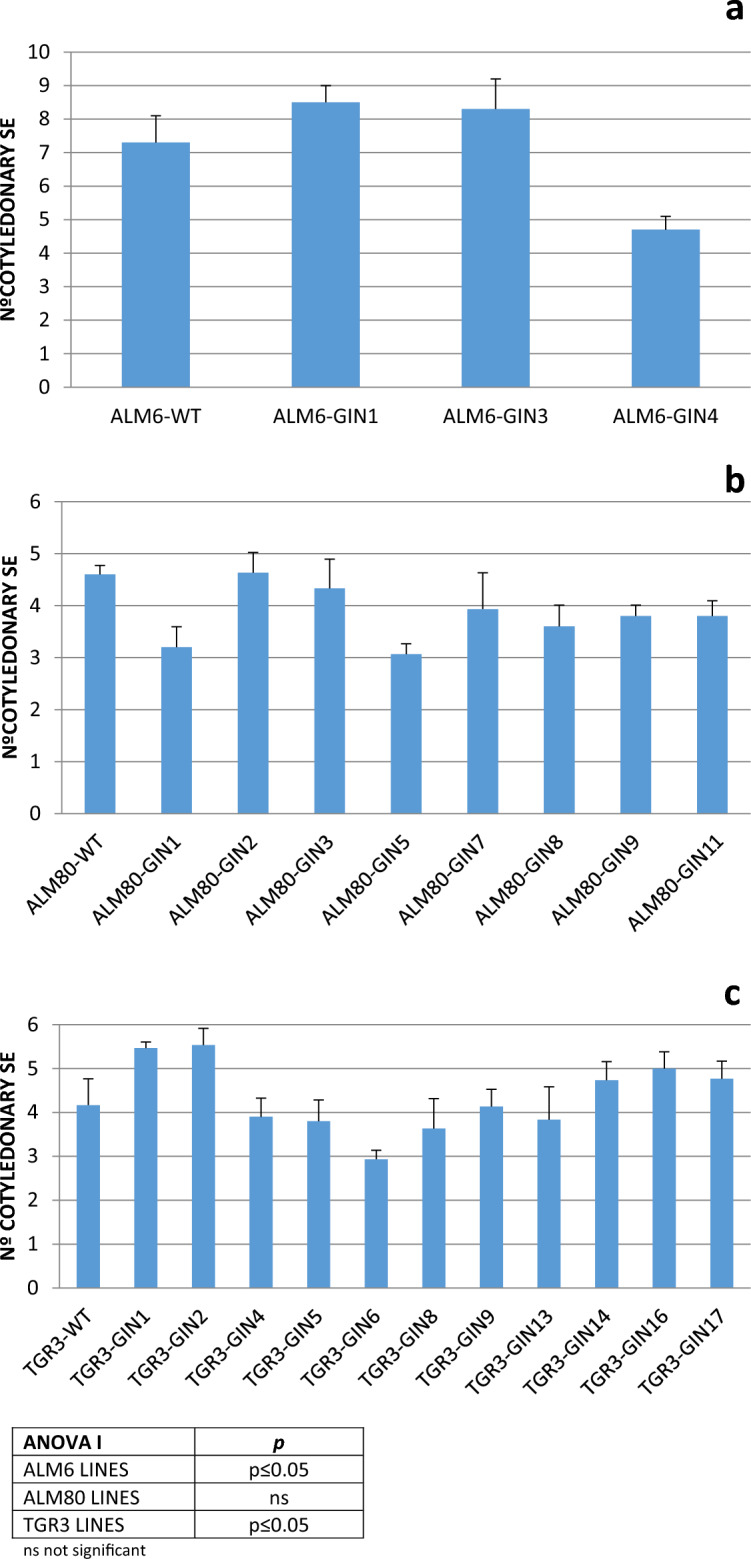
Production of somatic embryos cotyledonary stage from non-transgenic and transgenic lines generated from ALM6 (**a**), ALM80 (**b**) and TGR3 (**c**) genotypes. In each genotype, columns represent means ± error standard of 3 Petri dishes with 8 explants each. The statistical analysis of these data by ANOVA I is show in the table.

Once the different lines were established, the ability to form cotyledonary embryos larger than 5 mm (i.e. somatic embryos used for plant regeneration) was evaluated. In general, lines from ALM6 genotype showed more somatic embryos at cotyledonary stage than lines derived from genotypes TGR3 and ALM80 (Fig. 2). Among the three transgenic lines generated from genotype ALM6, the embryo production was significantly higher (*p* ≤ 0.05) in lines ALM6-GIN1 (8.5 se) and ALM6-GIN3 (8.3 se) (Fig. 2a). In genotype ALM80, the best results were obtained with lines ALM80-GIN2 (4.6 se) and ALM80-GIN9 (3.8 se) but without significant differences between the different lines generated (Fig. 2b). By contrast, significant differences (*p* ≤ 0.05) were observed between transgenic lines generated from TGR3 genotype and the embryo production was higher in lines TGR3-GIN1 and TGR3-GIN2 (Fig. 2c). Hence, these transformed lines from the 3 genotypes were selected for molecular analysis.

### Molecular analysis of transgenic lines

**Figure 3:**
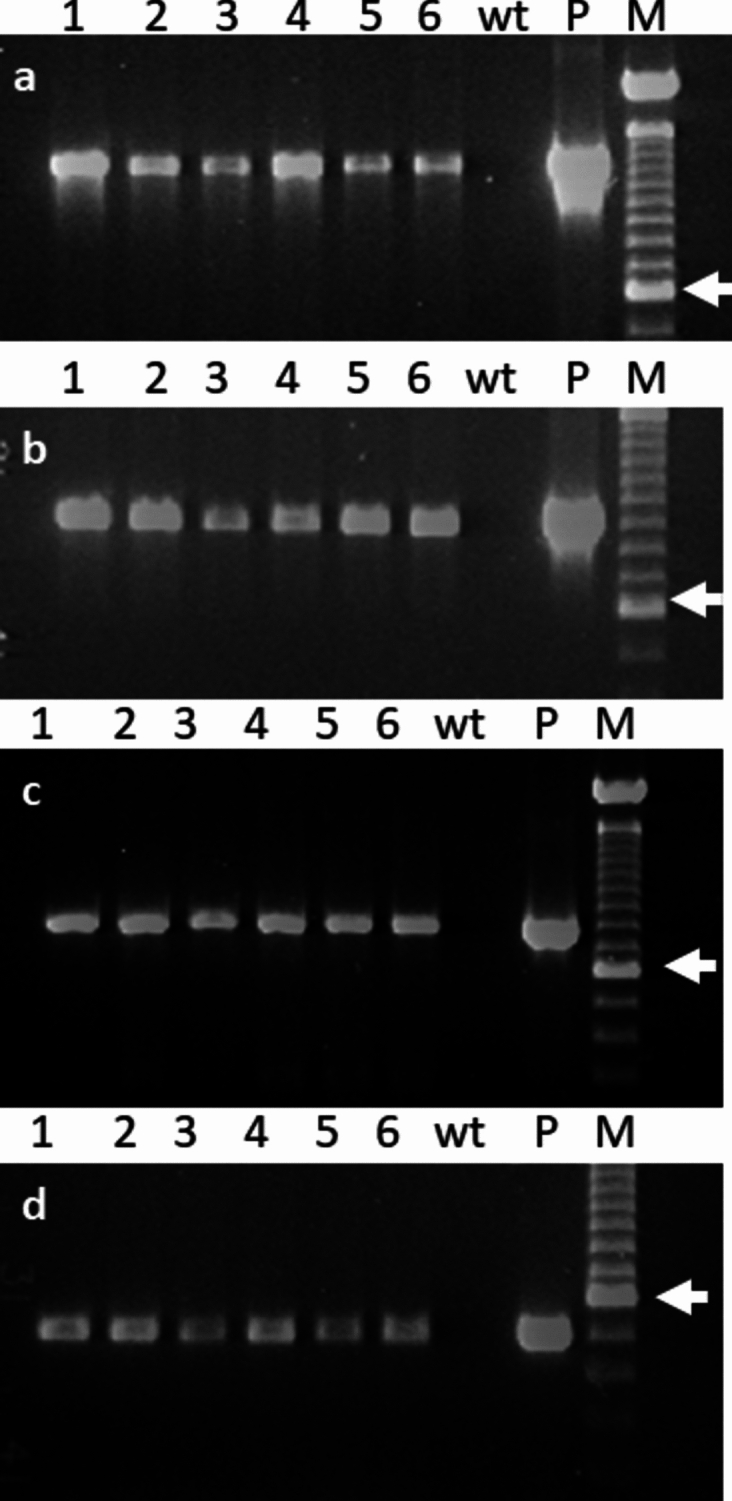
Confirmation by PCR amplification of the presence of *Cast_Gnk2-like* gene. Cast_Gnk2-like-R (**a**), Cast_Gnk2-like-F (**b**), GFP (**c**) and NPTII (**d**) fragments in the transformed cork oak lines. M: 600 bp molecular weight marker; P: plasmid; wt: non-transformed line; lanes 1–2: amplification of the transformed lines of ALM6-GIN1 and -GIN3; lane 3–4: amplification of the transformed line of ALM80-GIN2 and -GIN9; lanes 5–6: amplification of the transformed line of TGR3-GIN1 and -GIN2; arrows indicate 600 pb.

The presence of transgenes in the kan-resistant lines was confirmed by PCR analysis. The 472 bp of *NPTII* fragment, 740 bp of *GFP* fragment and 890 bp and 1227 bp of *Cast_Gnk2-like* fragments (i.e. the gene was amplified in both transcriptional senses; see Supplementary Information 1) were successfully amplified from all the transgenic lines and in the plasmid (positive control) confirming presence of the genes in the genome of somatic embryos (Fig. 3). No amplification product was observed in non-transgenic control somatic embryos of the three cork oak embryogenic lines evaluated (Fig. 3).

*Cast_Gnk2-like* copy number in the transformed lines was estimated in somatic embryos with the amplification of a CaMV35S promoter fragment (Table 2). The correlation coefficient of the standard curve that plots the C_T_ with the copy number was 0.89, despite the high efficiency obtained in the qPCR reactions (see Supplementary Information 1). The value of R^2^ may reflect the high variation in the detection of fewer copies, close to the limit of equipment detection. The highest number of transgene copies (5) was obtained for ALM6-GIN1, followed by TGR3 lines (3), ALM6-GIN3 (2) and ALM80 lines (1) (Table 2).

**Table 2:** Estimated copy number in cork oak somatic embryogenic lines transformed with *Cast_Gnk2-like* gene.

Line	C_T_ Mean	C_T_ Standard deviation	Estimated copy number ^1^
ALM6-WT	25.47	0.83	0
ALM6-GIN1	18.60	0.83	5
ALM6-GIN3	20.76	0.55	2
ALM80-WT	26.30	0.77	0
ALM80-GIN2	21.63	0.20	1
ALM80-GIN9	21.27	1.38	1
TGR3-WT	25.81	1.51	0
TGR3-GIN1	20.02	0.79	3
TGR3-GIN2	20.25	0.89	3

^1^Values from the correlation between the quantity of plasmid and the number of copies in cork oak, as described in Material and Methods.

**Figure 4:**
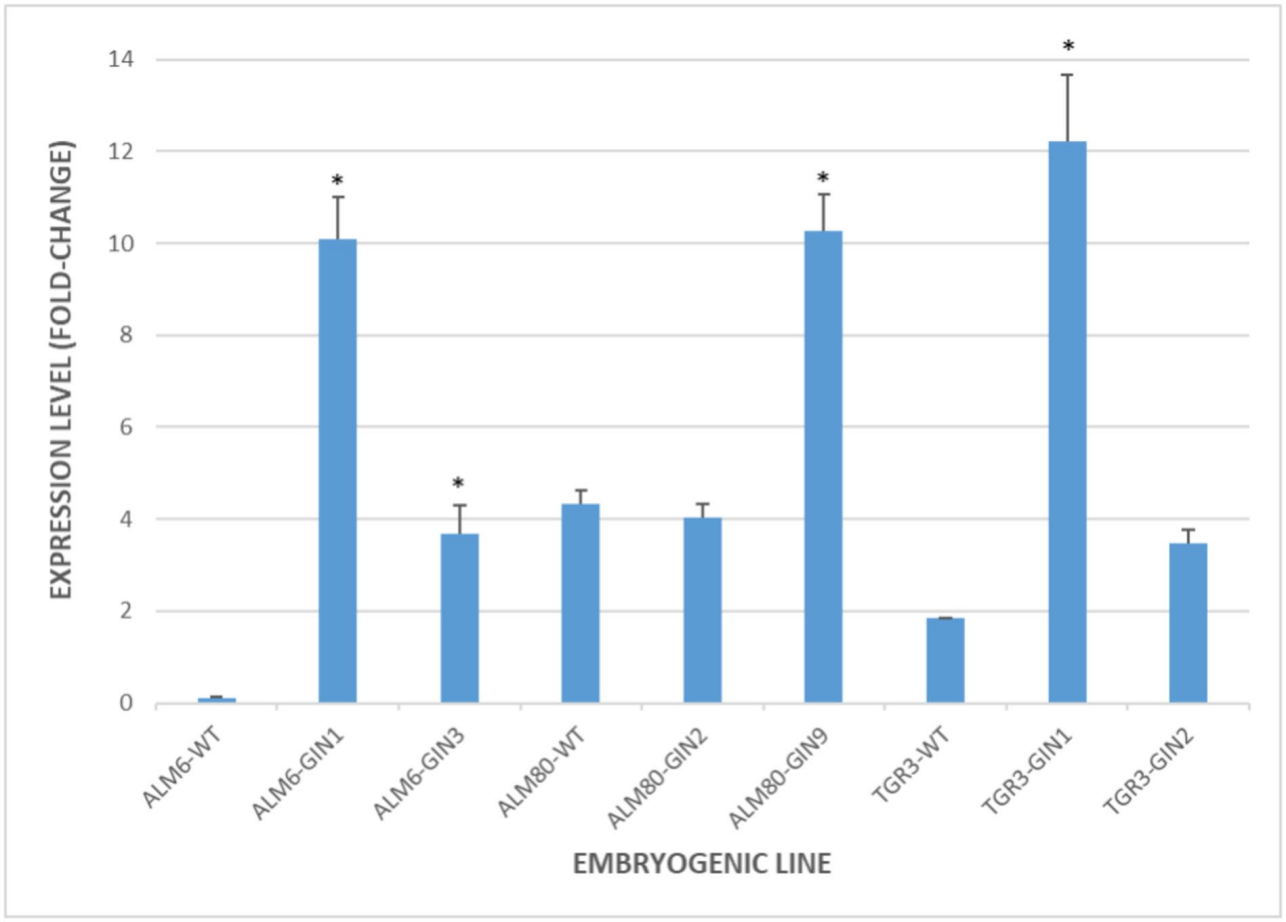
Expression analysis of *Cast_Gnk2-like gene* in somatic embryos of cork oak. Expression level in transformed lines and non-transformed lines (WT) was normalized to *Q. suber* Elongation factor 1-α and Tubulin reference genes. Bars are mean ± standard error (n = 3). Asterisks indicate significant differences in the expression when compared to non-transformed lines with *p* < 0.05 (Tukey HSD).

Transgene expression in each transformed line was compared to corresponding non-transformed genotypes in somatic embryos (Fig. 4). *Cast_Gnk2-like* oligos used in qPCR were based on the coding sequence of the original species, *Castanea crenata*. *Q.suber* and *C. crenata* are of the *Fagaceae* family, and when a BLASTn is executed in GenBank with *Cast_Gnk2-like* ORF as Query, the orthologous gene *Q. suber* cysteine-rich repeat secretory protein 38-like (Supplementary Information 2). Therefore, endogenous and transgenes transcripts may be amplified in the same reaction. But *Cast_Gnk2-like* had CaMV35S as the promoter, which has a constitutive action, so an overexpression of the transgene in transformed lines and an expression level higher than in the non-transformed lines was expected. This was true and significant for ALM6, TGR3-GIN1, and ALM80-GIN9 transformed lines (Fig. 4). ALM80-WT and TGR3-WT showed evident basal expression levels (4.3 and 1.8 respectively), and the transformed lines ALM80-GIN2 and TGR3-GIN2 didn’t show an overexpression of the transgene.

### Plant regeneration of transgenic lines

Plant regeneration ability was assessed in transgenic lines exhibiting elevated production of cotyledonary embryos compared to their respective non-transformed counterparts. Cotyledonary embryos were stored for two months at 4 °C under darkness conditions (see Supplementary Information 3) and subsequently they were cultured on germination medium. Regeneration was evaluated as simultaneous development of shoot and root (i.e. plant conversion), or as the development of only root (see Supplementary Information 3). After 8 weeks in the germination medium, only root development was observed in all tested cork oak lines, indicating uniformity in this aspect across the lines (Table 3). However, differences in the values of all evaluated parameters were observed among the lines (Table 3). The higher conversion frequencies were observed in line ALM6-WT and their corresponding transgenic lines with values ranging 83 to 92% (Table 3). Line TGR3-WT and their corresponding transgenic lines were the ones that showed the lowest conversion percentages (27–33%), whereas line ALM80-WT and their corresponding transgenic lines presented intermediate conversion values (23–53%). The quality of the plants evaluated in terms of root length, shoot length and number of leaves was higher in almost all transgenic lines compared to their wild-type counterparts.

**Table 3:** Plant regeneration performance with only root development and conversion into plantlets (root and shoot development) of different cork oak transformed and untransformed lines after 8 weeks on germination medium.

Line	Root only		Conversion (shoot + root)			
	(%)	RL (mm)	(%)	RL (mm)	SL (mm)	LN
*LINES ALM6*						
ALM6-WT	12.5 ± 6.2	45.0 ± 2.2	83.3 ± 7.4	71.4 ± 9.3	17.5 ± 0.9	3.7 ± 0.4
ALM6-GIN1	12.5 ± 9.7	80.0 ± 0.0	83.3 ± 9.1	119.4 ± 9.0	15.8 ± 1.5	7.9 ± 1.0
ALM6-GIN3	4.2 ± 3.2	68.0 ± 0.0	91.7 ± 3.7	107.3 ± 13.4	20.8 ± 1.7	6.6 ± 0.6
*ANOVA I*	ns	ns	ns	p ≤ 0.05	ns	ns
*LINES ALM80*						
ALM80-WT	30.0 ± 8.7	31.2 ± 5.0	53.3 ± 8.7	44.3 ± 5.3	14.2 ± 2.4	6.3 ± 0.6
ALM80-GIN2	53.3 ± 11.9	61.0 ± 16.0	23.3 ± 7.6	74.0 ± 19.5	16.9 ± 3.2	6.6 ± 0.3
ALM80-GIN9	56.7 ± 12.1	38.6 ± 4.1	30.0 ± 9.9	34.9 ± 6.6	16.5 ± 1.7	8.1 ± 0.7
*ANOVA I*	ns	ns	ns	ns	ns	ns
*LINES TGR3*						
TGR3-WT	70.0 ± 10.7	40.9 ± 10.7	30.0 ± 12.8	50.0 ± 15.8	10.5 ± 1.7	5.2 ± 0.6
TGR3-GIN1	53.3 ± 5.6	63.3 ± 11.9	33.3 ± 8.2	57.0 ± 16.8	15.3 ± 2.4	6.7 ± 1.3
TGR3-GIN2	66.7 ± 10.5	49.4 ± 8.0	26.7 ± 12.1	114.9 ± 15.1	15.6 ± 0.3	5.3 ± 0.9
*ANOVA I*	ns	ns	ns	ns	ns	ns

Each value represents the mean ± standard error of 5 replications with 6 explants in each replicate. *RL* Root length, *SL* Shoot length, *LN* Leaf number, *ns* not significant.

GFP expression was also detected in roots and leaves isolated from transgenic plants (Fig. 1d,e). As expected, no green fluorescence was observed in roots and leaves isolated from non-transformed regenerated plants (Fig. 1d,e). No phenotypic differences were found relativeto untransformed control plants, indicating that the GFP gene does not provoke deleterious or toxic effects on cork oak cells (Fig. 1f).

### Tolerance assay to Pc

**Figure 5:**
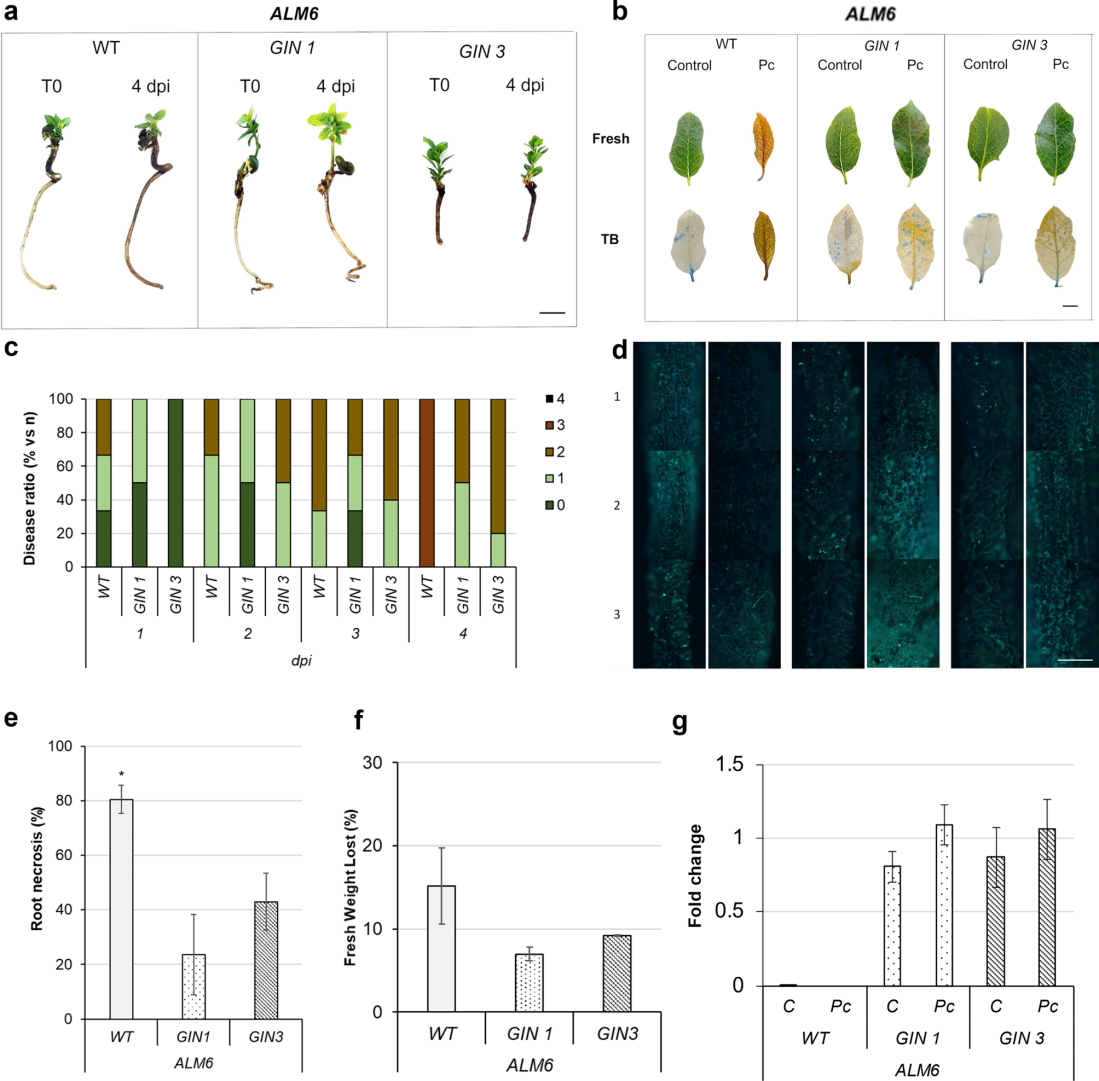
Disease symptoms of *Quercus suber* responding to *Phytophthora cinnamomi* Rands (Pc). ALM6-WT plants and in transgenic ALM6-GIN1 plants were inoculated with zoospores (10^7^ zs/ml). **a** Symptoms on explants at time “0” (T0), and after 4 days post-inoculation (4 dpi). Bar: (1 cm). **b** Detail of cell death symptoms on fresh leaves and stained with Trypan Blue (TB). Bars: (1 mm). **c** Kinetic of disease symptoms expressed in percent related to the total number of explants per treatment (n = 6), at 1, 2, 3 and 4 dpi, where: 0. No symptoms, 1. Leaf chlorosis and light necrosis on roots, 2. Apparent necrosis on leaves and light necrosis on roots, 3. High necrosis on leaves and roots, 4. Decayed explant. **d** Callose deposition on roots (1, differentiation, 2, elongation, and 3, meristematic area), detected by Aniline Blue staining on WT (left) and transgenic ALM6-GIN 1 (middle), and ALM6-GIN 3 (right), explants, inoculated (left panels) with zoospores or non-inoculated (right panels). (100 × magnification). Bar: (1 mm). **e** Root necrosis percentage on explants at 4dpi. **f** Fresh weight lost expressed in percentage related to controls at 4dpi. **g** Quantification of the expression of *Cast_GNK2-like* gene in WT and transgenic plants infected (Pc) and non-infected (C), data were normalized with *β-Tubulin* reference gene. The * in **e** denotes a statistically significant difference using variance check (*P*-value ≤ 0.05) and Tukey HSD test.

To determine under controlled axenic conditions, if the expression of the *Cast-Gnk2-like* gene might improve tolerance to Pc, plants of ALM6-WT, ALM6-GIN1 and ALM6-GIN3 were inoculated with zoospores (see methods section below). Root necrosis and disease symptoms were evident on wild type plants relative to transformed plants in all assays performed (Fig. 5a,b). At four days post-inoculation, wild type plants decayed while transgenic plants had lighter root necrosis (Fig. 5c). After 4 days, all non-transgenic plants die and the tolerance experiments were concluded to obtain enough non-necrotized tissue to achieve RNA from the roots. At this time, callose deposition was higher in transgenic plants when compared to non-transgenic ones (Fig. 5d; Supplementary Information 4). Root necrosis was in accordance to the loss of fresh weight observed, being higher in wild-type plants in comparison to transformed plants (Fig. 5e,f). The transcriptional levels of *Cast-Gnk2-like* gene were quantified using qPCR, revealing a correlation between the expression of the *Cast_Gnk2-like* gene and increased plant tolerance to Pc in ALM6 plants compared to wild types, as illustrated in Fig. 5g.

## Discussion

Oak decline syndrome, mainly caused by *P. cinnamomi*, has provoked serious damage in cork oak populations and therefore considerable economic losses. To date, cultural practices and chemical methods to control Pc were scarcely effective^38^. Additionally, fungicides are environmentally detrimental and expensive^39^. In this context, genetic transformation offers an alternative not only generating transgenic plants with enhanced disease tolerance, but also to provide us a method to explore the function of putative genes involved on the response mechanisms of plants to pathogens^40,41^. Although transgenic strategies have an evident potential to enhance cork oak disease tolerance, to date, only a gene with antifungal activity has been evaluated in this species^25^. In the present study, the first challenge was the overexpression of *Cast_Gnk2-like* gene, a novel type of antifungal protein, isolated from *C. crenata* transcriptome by using somatic embryos as target material. An efficient in vitro regeneration system is imperative to produce transgenic plants^42^. In this regard, somatic embryogenesis is considered the ideal system to address genetic transformation in woody plants, especially in highly recalcitrant species^43,44^. In the present report, genetic transformation was achieved in the three embryogenic lines evaluated although results obtained here showed that transformation frequencies with *Cast_Gnk2-like* gene varied widely with the genotype of embryogenic lines (2.5–9.2%). The scarce protocols previously described in the literature for overexpression of interesting genes in cork oak were also highly dependent on the genotype of the embryogenic lines. In line with our current investigation, Alvarez et al. ^45^ documented significant variances among the six embryogenic lines during transformation with marker genes. Similarly, Cano et al. ^25^ identified the influence of genotype on the transformation process using the same embryogenic lines featured in this report, particularly when overexpressing the *CsTL1* gene, encoding a thaumatin-like protein. As in this previous report, genotype TGR3 produced the best transformation frequencies, which reinforces the role of genotype in transformation. The effect of genotype on transformation rates was also mentioned for other members of *Fagaceae* family such as chestnut^46–50^, pedunculated oak^51^ and holm oak^52^. Although transformation rates are affected by the genotype, the values obtained in our report were higher than those described for other species using the same gene. Previously, we achieved transformation values of 2.5% using nodular embryogenic structures of holm oak transformed with *Cast_Gnk2-like* gene^37^. McGuigan et al.^50^ obtained 14 putative embryogenic lines expressing the *Cast_Gnk2-like* gene. However, only 3 were confirmed as containing the T-DNA insertion using RITA® Temporary Immersion Bioreactors to transform American chestnut somatic embryos.

As for holm oak, in the present study, *GFP* and *NTPII* genes were also successfully introduced and expressed in somatic embryos of the three embryogenic lines evaluated. This double selection with kan and GFP provided, respectively, a good selection and excellent visual screening of transgenic tissues in cork oak, as high levels of GFP expression were observed in both somatic embryos and in transgenic plants. Also, in both tissue types, non-toxic or deleterious effects were detected. This result points out that transgenic cork oak embryos could be selected using GFP expression alone even though the addition of kanamycin could improve the efficiency. This efficiency explains why GFP has replaced β‐glucuronidase (GUS) method as reporter procedure in transgenic plants due to its rapid detection, its high sensitivity, which does not harm the plant in which it is expressed and allows live visualization of tissues, using a UV light and without tissue destruction^53,54^.

The estimated *Cast_Gnk2-like* gene copies in transgenic lines varied from 1 to 5. Similarly, Alvarez et al.^24^ found variations from 1 to 4 copies of the inserted *phosphinothricin acetyl transferase* among transformed cork oak lines. A relationship between the number of transgenes and the expression level was not found. Other factors besides the copy number influence the expression level of the transgenes. One of them, the promoter CaMV35S, was used to confer high and constitutive expression of *Cast_Gnk2-like*. In effect, transgenic lines with different levels of *Cast_Gnk2-like* overexpression were obtained for all the somatic embryo genotypes, which was an important achievement for the *Cast_Gnk2-like* functional analysis approach. CaMV35S-driven transgene expression can be highly variable in the different transformants with the same construct^55^. Other factors that influence transgene expression are the position and the integration of the T-DNA in the genome. For instance, if multiple T-DNAs are integrated in one locus, they can be organized as direct or inverted repeats, resulting in different levels of expression^55^.

Remarkably, gene expression levels observed in non-transformed ALM80 and TGR3 indicate a high basal level of a endogenous *Q. suber* ginkbilobin2-like. The alignment of the aminoacid sequences of such endogenous protein with Cast_Gnk2-like show a high similarity between them (95%, Supplementary Information 2). In common there is a predicted signal peptide for secretion and 2 conserved cysteine-rich domains (C-X8-C-X2-C). The second domain is equal, although in the first domain there is one dissimilar aminoacid (lysine for the endogenous protein, threonine for *C. crenata*). The 2 aminoacids have different R-groups, lysine has an electrically charged side chain and threonine has a polar uncharged side chain. Could this difference contribute to confer different resistance levels to *Q. suber* and *C. crenata*? Our results indicate that the low basal expression level of the gene in non-transformed ALM6 is not sufficient to confer tolerance to *P. cinnamomi*. Additional assays will be necessary to determine if *Q. suber* ginkbilobin2-like has a relevant role against the pathogen.

In addition to *Cast_Gnk2-like* gene overexpression in *Q. suber* somatic embryos, a second challenge was to generate viable plants from transgenic somatic embryos to use for tolerance experiments. The ability to regenerate complete plants from transformed cotyledonary-stage embryos is one of the main bottlenecks in woody plants as it has been mentioned in previous studies, as in the genetic transformation of somatic embryos of European chestnut^48^ or pedunculate oak^51^. Nevertheless, in the present report suitable conversion percentages have been obtained, especially in transgenic lines derived from ALM6 genotype. Moreover, the plants obtained showed a good quality which allowed us to have adequate material for the evaluation of their tolerance to *P. cinnamomi*.

The selection criteria for conducting tolerance analysis in transgenic lines often hinge on the expression levels of the transgene. However, determining the optimal criterion remains a subject of debate. Some studies advocate for evaluating transgenic lines with higher protein expression levels, arguing that elevated expression increases the likelihood of achieving tolerance against pathogens^25,56–58^. Conversely, other researchers have failed to observe a clear correlation between tolerance and gene expression levels^59^. In our study, we opted to select embryogenic lines for tolerance analysis based on their high conversion rates and root length.

Overexpression of the *Cast_Gnk2-like* gene potentially confers improved tolerance to *P. cinnamomi* in cork oak plants using an in vitro test. Evaluating biotic and abiotic tolerance in plants poses significant challenges, particularly in woody species^60^. In vitro testing has emerged as a valuable method for assessing tolerance, offering the advantages of handling a larger number of specimens in a confined space and precise control over environmental conditions^61^. While in vitro tests have predominantly been employed for assessing tolerance to abiotic stress, there are instances of their application in evaluating tolerance to biotic stress, albeit primarily in herbaceous species such as carnation^62^, *Arabidopsis*^63^, and rice^64^. In our study, we conducted an in vitro tolerance test by inoculating cork oak plant roots with a standardized stock of zoospores under controlled conditions to minimize additional variability that may arise from using mycelium fragments ^65^, building upon prior methodologies^66–68^. Zoospore inoculation offers a faster and precise mimicry of *P. cinnamomi* infection in natural conditions. Our in vitro assays revealed that plants overexpressing the *Cast_Gnk2-like* gene exhibited reduced disease symptoms compared to untransformed plants, with approximately half the root necrosis observed in wild-type plants, along with increased callose deposition. Callose deposition is recognized as a key stress response in plants^69^, with its role in defense responses being contingent upon the pathogen's mode of infection. For instance, callose deposition in avocado roots' parenchyma and cortex is crucial for tolerance by impeding root zoospore colonization^70^, a phenomenon observed in maize as well ^71^. Callose deposition at plasmodesmata serves to impede cell-to-cell spreading induced by viruses and blast fungus^72^, restricting the spread of *Phytophthora brassicae* infection^73^ and potentially limiting cell-to-cell trafficking, thus safeguarding the plant host. However, mutants of the Powdery Mildew Resistance *(PMR4)* callose synthase gene exhibited enhanced tolerance to oidium^74^, suggesting a context-dependent role of callose. Cahill and Weste^75^ demonstrated in over 30 species, including both forest and non-forest species, that *P. cinnamomi* infection induced by zoospore inoculation led to callose deposition and papillae production around germ tube entry, exclusively in tolerant hosts, while being absent in susceptible ones. Our results, obtained under controlled conditions, indicate that callose deposition is induced in transgenic lines post-infection, whereas it was scarcely detectable in wild-type roots, the latter of which exhibited near-total root death. We found a positive correlation between callose deposition induction and the overexpression of the *Cast_Gnk2-like* gene, suggesting that this gene alters callose deposition patterns, thereby enhancing plant tolerance. Contrasting with inoculated plants, callose deposition in non-inoculated transgenic plants remained lower than in wild-type plants, indicating that callose deposition is modulated by gene overexpression. While overexpression of stress-related genes may contribute to root callose deposition, the correlation with increased tolerance is not always straightforward^76^, likely dependent on the pathogen's infection mechanism. On other hand, besides the role in stress response, callose also plays a crucial role in plant development, e.g. in cell plate formation during cell division (Chen and Kim^77^). In wild type non-inoculated plants, callose deposition can be interpreted as an adaptation to the in vitro inoculation, focusing on growth and cell division. In contrast, wild type inoculated plants do not exhibit callose signals because the cells have decayed due to infection. Our findings suggest that *Cast_Gnk2-like* gene overexpression in *Q. suber* contributes to callose deposition in response to *P. cinnamomi*, though the molecular underpinnings necessitate further investigation.

To confirm the high levels of *Cast_Gnk2-like* gene expression in ALM6-GIN 1 and GIN 3 lines during inoculation assays, qPCR was performed. As anticipated, both lines exhibited consistently high gene expression levels throughout infection, with a slight non-significant increase observed in infected plants. No significant differences were observed between the two lines, likely due to the already high levels of overexpression in both.

To date, only two studies have investigated the overexpression of a *GNK-2* gene, and in line with our results, both reported enhanced tolerance. For instance, overexpression of *ginkbilobin2-1 (GNK2-1)* from *Ginkgo biloba* seed kernels in transgenic cucumber plants inhibited fungus growth when infected with *Fusarium oxysporum*^32^. Similarly, overexpressing the *Cast_Gnk2-like* gene in nodular embryogenic structures of holm oak significantly increased the survival period during infection with Pc^37^.

In intragenesis and cisgenesis procedures, genes transferred contain the same gene pool as in conventional breeding^78^. As *Cast_Gnk2-like* gene has been identified in Japanese chestnut, we would be overexpressing a gene from the same family as the cork oak, and consequently the plants obtained will not be strictly transgenic, being able to call intragenic if *Cast_Gnk2-like* gene was driven by a constitutive plant promoter (i.e. Ubiquitin 10 (UBQ10) promoter of *Arabidopsis*). In the last decade, the scientific community has showed great interest in promoting less stringent regulations for cisgenic/intragenic plants^79–81^. Several surveys and focus group interviews in the USA, Europe and other countries show that intragenic and cisgenic plants are more acceptable than strict transgenic plants, encouraging their use^78,81^. Hence, less rigorous plant transgenic regulations could reduce the costs and boost their commercial use^78^.

## Material and methods

### Plant material

For the genetic transformation, three embryogenic lines (ALM6, ALM80 and TGR3) induced from leaves derived from centenary trees were used^20^. These embryogenic lines have been maintained by secondary embryogenesis, with subcultures every 6 weeks, in proliferation medium (for more information see Supplementary Information 5). The embryogenic lines were cultured in a growth chamber under a 16-h photoperiod (provided by cool-white fluorescent lamps at a photon flux density of 50–60 µmol m^-2^ s^-1^) and 25 °C light/20 °C dark temperatures (standard conditions).

### Transformation vector and bacterial strain

The *Cast_Gnk2-like* gene, which encodes a ginkbilobin-like protein, was cloned into the pK7WG2D vector^82^ under the CaMV35S promoter, using the Gateway system (Invitrogen, USA), as described Corredoira et al.^83^. The plasmid also includes the neomycin phosphotransferase gene (*NPTII*), and the green fluorescent protein (*EGFP*) reporter gene, used as a visual indicator of transformation. The *NPTII* gene is regulated by the promoter of the nopaline synthase (*Nos*) gene, whereas the *EGFP* gene is controlled by the promoter of the rol root loci D (rolD) gene. The vector with the three genes was called pK7WG2D-GIN (see Supplementary Information 6) and was transferred to the *Agrobacterium tumefaciens* strain EHA105^84^ using the freeze–thaw method defined by Xu and Li^85^. The resulting strain, named, EHA105pK7WG2D-GIN, was employed in all transformation experiments performed in the present report.

### Somatic embryo transformation

For transformation, groups of 2 or 3 somatic embryos (4–7 mg; se) in the globular or torpedo stage of the three embryogenic lines were isolated 4 weeks after the last subculture and were precultured in 24 h in Petri dishes with preculture medium under standard conditions. The precultured explants were immersed in the infection medium with EHA105pK7WG2D-GIN which was prepared as described in Corredoira et al.^83^. After 30 min with gentle agitation, infection medium was removed by filtration and the explants were transferred to coculture medium and cocultured for 5 days in darkness at 25 °C. Bacteria were eliminated by immersion of explants for 30 min in a washing solution consisting of sterile water with carbenicillin (CB; 300 mg/L). The washing solution was removed by filtration and the explants were transferred to selection medium that consisted of proliferation medium supplemented with 300 mg/L CB and 125 mg/L of kanamycin (kan) (for more information see Supplementary Information 5). The embryos were maintained in standard conditions, with periodic subcultures every two weeks to this selection medium.

In each embryogenic line, 120 explants were used (6 Petri dishes with 10 explants and the experiment was repeated twice) resulting in a total of 360 explants. Additionally, for each line 20 explants that were not infected and that were cultured on proliferation medium without antibiotics (control positive) and with antibiotics (negative control).

### Selection and maintenance of transgenic lines

After 10 weeks on selection medium, explants that showed signs of growth were transferred to proliferation medium (Fig. 1a) supplemented with a higher concentration of kan than in the selection medium (150 mg/L) and were cultivated for 4 more weeks, in order to increase the selective pressure and confirm its resistance to kan. After 14 weeks from the beginning of the transformation, the percentage of kan-resistant (defined as the percentage of initial explants that are resistant to kan) and the transformation efficiency (defined as the percentage of initial explants that showed fluorescence (GFP +) were determined. The fluorescence was observed using a Leica M205 FA magnifier (Germany), equipped with a 200-W bulb, and a specific filter for fluorescence, with 470/40 × nm excitation and 525/50 nm emission. The images were taken with a Leica DSC7000T camera (Germany). From each GFP + explant, a single somatic embryo at cotyledonary stage was isolated, and each embryo was multiplied by secondary embryogenesis in selection medium to establish the different transgenic embryogenic lines. During establishment period, GFP expression was also verified periodically to rule out the appearance of chimeras. After approximately four months, when the multiplication of GFP + lines was obtained, they were transferred to medium without antibiotic, to eliminate selective pressure and increase multiplication rates. To test the proliferation ability, the number of cotyledonary somatic embryos > 5 mm produced by explant was evaluated and compared with those of the corresponding non-transformed (control) embryogenic line.

### Molecular analysis of transgenic embryogenic lines

#### Gene analysis presence in embryogenic lines

Total genomic DNA was isolated from 100 mg of cotyledonary somatic embryos of kan-resistant embryogenic lines and non-transformed embryogenic lines (WT) using the Kit REALPURE (Durviz, Spain) following the instructions of the manufacturer. PCR was performed to amplify a specific DNA sequence in transgenic lines corresponding to the *GFP*, *NTPII* and *Cast_Gnk2-like* genes (see Supplementary Information 1). A 25 µl PCR reaction was prepared containing 250–500 ng of genomic DNA, 15 µM of primers, 0.2 μL Taq DNA Polymerase (Qiagen, Germany), 1μL MgCl_2_, and 2.5 mM of dNTPs. Plasmid DNA was used as a positive control, whereas DNA from a non-transformed control somatic embryos and distilled water served as negative controls. PCR products were separated in 1.5% (w/v) agarose gels (Ultra Pure Agarose, Invitrogen, USA) in 1 × TBE buffer at 90 V. After electrophoresis, gels were stained with 1% de RedSafe (iNtRON Biotechnology, Korea) and visualized under the UV light.

#### Gene number copy analysis in embryogenic lines

The number of *Cast_Gnk2-like* copies inserted in the transformed lines was estimated by quantitative real-time PCR (qPCR), similarly as described in^37^. In brief, DNA was extracted as in the previous section, and three biological replicates per genotype were prepared. Specific oligos for the CaMV35S promoter were used in the reactions (see Supplementary Information 1). A standard curve was established as in Song et al.^86^, by mixing the plasmid used for transformation with non-transformed genomic DNA. Using the value of *Q. suber* genome size reported by Ramos et al.^87^, we calculated the quantity of plasmid needed to be mixed with non-transgenic DNA to simulate 1, 2, 5 and 10 copies of transgene. qPCR cycling with a subsequent step for the melting curve was performed as in Mallón et al.^88^. The qPCR experiments included no template controls, two technical replicates and two repetitions. The copy number in each sample was estimated making the correspondence between the C_T_ and the mocked copies of the transgene.

#### Gene expression analysis in embryogenic lines

The expression of *Cast_Gnk2-like* gene was analyzed by qPCR. Total RNA was extracted from non-transformed and putatively transformed lines using somatic embryos at early cotyledonary stage and a protocol with CTAB (hexadecyltrimethylammonium bromide buffer) based on^89,90^. Three biological replicates were considered from three different embryo clumps, for both non-transformed and transformed lines. Once RNA was extracted, it was treated with the RNase-free DNase Set (Qiagen, Hilden, Germany) to eliminate possible DNA contamination. The Qiagen RNeasy Plant Mini Kit (Qiagen, Hilden, Germany) was then used for RNA Cleanup according to the manufacturer’s instructions. The stability of the extracted RNA was verified by observing a double band in gels of 1% agarose, and the concentration was quantified with a Thermo Scientific™ NanoDrop™ One Microvolume UV–Vis Spectrophotometer (Thermo Fisher Scientific, Waltham, MA, USA). Total RNA (2 μg) was used as the template for reverse transcription with RevertAid H Minus Reverse Transcriptase (Thermo Fisher Scientific, Waltham, MA, USA) and primed with an oligo(dT) primer. Specific primers for *Cast_Gnk2-like* are described in Supplementary Information 1. 20 ng of cDNA were used per reaction in a 15 μL final volume using 7.5 μL of NZYSupreme qPCR Green Master Mix (2x) (NZYtech, Carnide, Lisboa, Portugal). A final concentration of 0.2 μM of each primer was used in a CFX96 Touch Real-Time PCR Detection System (BioRad, Hercules, CA, USA). Reactions started with a denaturation step at 95 °C for 10 min followed by 40 cycles of denaturation at 95 °C for 15 s and annealing temperature for 30 s. Each set of reactions included a no-template control and three technical replicates. Dissociation curves were used to analyze non-specific PCR products. To normalize expression data, *Elongation factor 1-alpha* (EF1α)^91^ and *β-Tubulin* (TUB)^92^ were used (Supplementary information 1). Gene expression was calculated using the Pfaffl method^93^.

### Plant regeneration from transgenic lines

For plant regeneration from cork oak somatic, previously defined procedure^94^ was used. Briefly, somatic embryos at cotyledonary stage (≥ 5 mm) were isolated from 6-week cultures of non-transformed lines and the transformed lines with the best proliferation rates. Embryos were transferred to 100 ml glass jars with plastic lids with 30 ml of proliferation medium and were stored at 4 °C in darkness. After two months, the embryos were transferred to 500 ml glass jars with 70 ml of medium germination (for more information see Supplementary Information 5). After 8 weeks of culture under standard conditions the following parameters were determined: the percentage of embryos that only developed roots ≥ 5 mm, and the percentage of embryos that developed a complete plant, as well as the length of the root (mm) and the shoot (mm) and the number of leaves per plant. For each WT line and their respective transgenic lines 36 somatic embryos (6 se per jar) were cultured.

### In vitro* tolerance assays*

#### Plant incubation conditions

Plants derived from germination of somatic embryos as described above were transferred from agar to plastic sterile tubes containing 1 ml of sterilized water and they were incubated in an Aralab chamber (Lisbon, Portugal), at 50% humidity (v/v), temperature of 24 °C during the day and 18 °C during the night, with a 16-h light/8-h dark photoperiod and light intensity of 150 µE. m^-2^ per second for all experiments. They were maintained in these conditions for a week.

#### Plant infection

The explants from agar tubes medium are incubated in an Aralab chamber (Lisbon, Portugal), at 50% humidity (v/v), temperature of 24 °C during the day and 18 °C during the night, with a 16-h light/8-h dark photoperiod and light intensity of 150 µE.m^-2^ per second for all experiments for a week, before to be transferred to new sterile culture tubes DeWit® (Sgl, Barcelona, Spain), containing 1 ml of sterile water and maintained for an additional two days for further acclimation. *Phytophtora cinnamomi Rands* was kindly provided by TRAGSA (Maceda nursery, Orense, Spain) and isolated by the Center for Research and Technology of Extremadura (CYCYTEX), located in Mérida, Spain.The *Phytophtora cinnamomi* mycelium culture was initially obtained from a fresh plate using previous describe methodology^66–68^. Then a zoospore stock was obtained, and saved at − 80 °C until use, following the previous optimized protocol^65^. He plants were divided into two groups: a control group and an inoculated group. The explants were inoculated into the sterile culture tubes DeWit® (Sgl, Barcelona, Spain), with *Phytophthora cinnamomi* zoospores stock at a final concentration of 10^7^ zoospores/ml or with sterilized water on controls.

#### Disease evaluation symptoms

The plant disease symptoms produced by *Phytophthora cinnamomi* Rands were considered following the standards approved for diagnostic protocols for regulated pests by EPPO council^95^. Those symptoms included principally root rot brown lesions followed by necrosis produced directly by the pathogen and secondary symptoms of decline producing leave chlorosis and later necrosis and dead. Disease symptoms were followed for 4 days using a modified infection criteria described by^96^ as followed: 0. No symptoms, 1. Leaf chlorosis and light necrosis on roots, 2. Apparent necrosis on leaves and light necrosis on roots, 3. High necrosis on leaves and roots, 4. Decayed explants.

Fresh weight (FW) was measured at 0 and 4 dpi, to calculate the fresh weight lost (FWL) Following the formula:

$$ {\varvec{FW}} \left( \% \right) = \frac{{\left( {FW 4 dpi \times 100} \right)}}{{FW\left( { T0} \right)}};\;\user2{FWL }\left( \% \right) = 100 \% - FW \left( \% \right) $$

Photographs of the explants were captured for assessing root necrosis. The quantification of root infection and necrosis involved measuring the total length of the infected root and the segments showing necrosis at 4 dpi using the software ImageJ. The calculations were performed using the following formula:

$${\varvec{R}}$$

$${\varvec{Root}}\, {\varvec{necrosis}} (\text{\%}) = \frac{(Length\, of\, infected\, area\, (cm)\, \times \, 100)}{Total\, length\, (cm)}$$

Necrotic lesions were measured by Trypan blue staining, performed as previously described^97^. Briefly, a Trypan Blue solution was prepared by combining 10 ml lactic acid (85% w:w), 10 ml phenol (TE balanced buffer, pH 7.5–8.0), 10 ml glycerol (≥ 99%), 10 ml distilled water, and 40 mg Trypan Blue, resulting in a final concentration of 10 mg/ml. Roots and leaves were stained for 20 min, rinsed with 100% ethanol over two days, and preserved in 60% glycerol until microscopic observation.

Callose deposition was detected by Aniline Blue staining applying an adapted procedure of the method described by^98^. Briefly, 0.1% aniline blue was prepared in 0.1 M of monosodium phosphate buffer (NaH_2_PO_4_·2H_2_O; pH = 9) and 2% glycerol (v/v). The root explants were immersed in the solution overnight in darkness. The stained samples were stored at 4 °C until microscopic observation.

Aniline blue was then detected using a DAPI/UV filter by fluorescence microscopy using a stereomicroscope (A292/21 Microscopy iScope IS.3153-PLFi/6 with Fluorescence—IS.3153- PLi/6,nEWF 10x/22, with Plan Fluarex PLFi, 4 × , 10 × , 20 × , 40 × , and 100 × oil lenses including fluorescence: Blue, Green, UV-DAPI, and Red filters, Microsercon SLU, Madrid, Spain) with a charge-coupled device (CCD) digital cooled camera (A292/21 Euromex 20 MP USB 3.0 with 1 inch CMOS sensor), to obtain digital photos. At least five photos were quantified per root area and treatment, considering taken on three different root areas of differentiation, elongation, and meristematic region and three plants per treatment. No callose was detected into apical root area.

### Quantitative real-time-PCR analyses in infected plants

Tissues derived from inoculated plants were frozen using liquid nitrogen and subsequently pulverized with a mortar and pestle to obtain a powder, which was then transferred into Eppendorf tubes. Total RNA extraction was conducted in two steps. In the first step, the method with CTAB buffer already described was employed. The second step involved purifying the RNA solution from the first step using the RNeasy Plus Mini Kit (QIAGEN, Germany) in accordance with the kit's protocol. cDNA was synthesized using NZY First-Strand cDNA Synthesis Kit according to the manufacturer’s protocol.

The qPCR experiments were performed using a fluorescence green Power Sybr Green PCR Master mix (Thermofisher, USA) with reactions at a final volume of 20 µl per well. Specific primers for *Cast-Gnk2-like* and for *Quercus suber* β-Tubulin as a referencegene are described in Supplementary Information 1. The successful specificity of both oligonucleotides amplified in *Quercus suber* was confirmed by getting a melting curve at the last step of corresponding qPCR reactions.

The *Cast_Gnk2-like* gene expression in the transgenic plants inoculated or non-inoculated was quantified using the 2 − λλCT method relatively to wild-type explants and using three biological triplicates to each condition and assay. Data points were compared using t-tests (see below). Three biological replicates were used from two different individuals for both control and inoculated entire plants, in ALM6-WT and their transgenic lines (ALM6-GIN1 and ALM6-GIN3), respectively.

### Statistical analysis

The SPSS 26.0 statistical package for Windows (Chicago, USA) was used to analyze the transformation and regeneration experiments. In transformation experiments, one-way analysis of variance (ANOVA I) was applied to determine the effect of genotype on genetic transformation of somatic embryos (Table 1) and the effect of genotype on the embryo production (Fig. 2) and plant regeneration (Table 3). In gene expression analysis–embryogenic lines, ANOVA I complemented by a Tukey HSD test was used for statistical significance analysis of the transgenic lines compared to the wild-type genotypes at *p* < 0.05 (Fig. 4). In the tolerance assay, the Stat Graphics Centurion XVI.II program (Stat Point Technologies, Inc., Warrenton, VA, United States) was used for all data analysis related to plant fresh weight lost, root necrosis and the expression of *Cast_Gnk2-like* gene. ANOVA I and Duncan’s mean comparison test were performed for all experiments and t-tests with a significance level of 0.05% (Fig. 5). Percentages were subjected to square-root transformation prior to analysis to normalize the data. Non-transformed data are presented in Tables and Figures. In the case of non-homogeneous variance, a non-parametric Kruskal–Wallis test was used.

## Conclusions

The results reported here are preliminary, but they indicate that *Cast_Gnk2-like* gene has an important role in the tolerance to *P. cinnamomi* and provides new insights to investigate the infection process of this oomycete. Furthermore, *Cast_Gnk2-like* gene is a promising tool for engineering plants for pathogen tolerance. However, the mechanism of this gene and its role in cork oak tolerance requires further research. While the plants generated in this study incorporate viral promoters and reporter/marker genes, the promising outcomes achieved with GFP selection and the incorporation of a gene isolated from a taxonomically close species suggest the potential for future cultivation of cork oak plants overexpressing this gene as enhanced and well-adapted varieties.

## Supplementary Information

Supplementary Information.

## Data Availability

All data generated or analyzed during this study are included in this article and its supplementary information file. The images of callose do not correspond to protein blots, corresponds to aniline blue staining so the images are just provided directly by the fluorescence microscope. Because of the long size of root explants, the amplification zoom corresponds to 10 × so the original images are obtained directly from it. We provided a link file with original images obtained into this work including replicates used for quantifications of snapshots corresponding to aniline blue staining. Original callose imagenes have been included.
