# Author Correction: Overexpression of Ginkbilobin-2 homologous domain gene improves tolerance to *Phytophthora cinnamomi* in somatic embryos of *Quercus suber*

**DOI:** 10.1038/s41598-024-72759-4

**Published:** 2024-09-12

**Authors:** Susana Serrazina, MªTeresa Martínez, Serine Soudani, Gonçalo Candeias, Marta Berrocal-Lobo, Pablo Piñeiro, Rui Malhó, Rita Lourenço Costa, Elena Corredoira

**Affiliations:** 1 Faculdade de Ciências, BioISI-Biosystems & Integrative Sciences Institute, Universidade de Lisboa, Lisbon, Portugal; 2 Misión Biológica de Galicia, Sede Santiago de Compostela, Consejo Superior de Investigaciones Científcas (MBG-CSIC), Avda Vigo S/N, 15705 Santiago de Compostela, La Coruña, Spain; 3 Centro Para La Biodiversidad y Desarrollo Sostenible (CBDS), ETSIMFMN, Universidad Politécnica de Madrid, Ciudad Universitaria S/N, 28040 Madrid, Spain; 4 Instituto Nacional de Investigação Agrária E Veterinária I.P., Oeiras, Portugal

Correction to: *Scientific Reports*10.1038/s41598-024-70272-2, published online 21 August 2024

The original version of this Article contained an error in the spelling of the author Gonçalo Candeias which was incorrectly given as Gonçlo Candeias.

The original Article has been corrected.

