# Supplementary material for: Overexpression of Ginkbilobin-2 homologous domain gene improves tolerance to Phytophthora cinnamomi in somatic embryos of Quercus suber
Source: Sci Rep. 2024 Aug 21;14:19357. doi: 10.1038/s41598-024-70272-2 (PMC11339267; doi:10.1038/s41598-024-70272-2)
Supplement: Supplementary file 1 — Supplementary Information. [file 41598_2024_70272_MOESM1_ESM.pdf]

Supplementary Information 1. Primers and amplification programs used in the present report.

GENE or PROMOTER	PRIMER NAME	PRIMER SEQUENCE (5′-3′)	PCR CONDITIONS	FRAGMENT AMPLIFIED (bp)	PURPOSE	Range of qPCR Efficiency (%) and R^2
NPTII	NPTII-F<br>NPTII-R	GTCATCTCACCTTGCTCCTGCC<br>AAGAAGGCGATAGAAGCGA	35 cycles:<br>94ºC x 30s<br>60ºC x 30s<br>72ºC x 42s	472	PCR analysis	-
GFP	EGFP-F<br>EGFP-R	CACCGGGGTGGTGCCCAT<br>CTAGTGGATCCCCCGGGC	40 cycles:<br>94ºC x 15s<br>56ºC x 30s<br>72ºC x 1min	740	PCR analysis	-
Cast_Gnk2-like-F[1]	T35S-R<br>GIN-D	AGGTCACTGGATTTTGGT<br>CTGCCACTAGCCGTTATGGT	35 cycles:<br>98ºC x 10s<br>56ºC x 30s<br>72ºC x 1min	890	PCR analysis	-
Cast_Gnk2-like-R[2]	p35S-D<br>GIN-R	GATCTAACAGAACTCGCC<br>CTGGTGCATTGAGCCAAACC	35 cycles:<br>98ºC x 10s<br>56ºC x 30s<br>72ºC x 1min	1227	PCR analysis	-
CaMV35S promoter	P35S-F<br>P35S-R	GGACGATTCAAGGCTTGCT<br>AGTCTTCACGGCGAGTTCT	Tm 58ºC	137	qPCR copy number	94-97<br>0.993-0.996
Cast_Gnk2-like	Cc_Gnk2-F<br>Cc_Gnk2-R	GGGGACCTAAAGCTTGACTCA<br>CATCGCAACAGTTGGGAAGTT	Tm 60ºC	129	Transgene expression for qPCR in somatic embryos and infected plants	100-76<br>0.990-0.993
β-Tubulin	Tub-F<br>Tub-R	CTGCGGTCGCTATGTTCCT<br>CCCTTGGCCCAGTTGTTTC	Tm 60ºC	147	Reference gene for qPCR	84-75<br>0.997-0.990
Elongation factor 1 -α	EF1α-F<br>EF1α-R	GTGCCGTCCTCATTATTGAC<br>CACGGGTCTGACCATCCTT	Tm 60ºC	75	Reference gene for qPCR	89-73<br>0.999-0.986

The presence of *Cast_Gnk2-like* gene was verified by PCR in both transcriptional senses employing the specific primers Cast_Gnk2-like-F and Cast_Gnk2-like-R. [1]This fragment includes T-35S region (See Supplementary Information 2). [2] This fragment includes CaMV35S region (See Supplementary Information 2). F: forward; R: reverse.

Supplementary Information 2

Program: **BLASTN** suite-2sequences (NCBI)
Query: Cast_Gnk2-like (dna) Length: 726
Subject: Qs_Gnk2-like (dna) Length: 726. NCBI ref. XM_024067951.1

Alignment:
Score:1208 bits(654), Expect:0.0,
Identities:702/726(97%), Gaps:0/726(0%), Strand: Plus/Plus

```
Query  1    ATGTTGAGCTCAAAATATATTTCTGTCAGCTTTCTATTACTCAGCCTCTCCCTCCATGCA  60
Sbjct  1    ......T...........T...................................T.....  60
Query  61   GTCAATTGTGCTGACCCATTATACCATTTTTGTTTTAGCCAAGAAAGCTACACTGCCACT  120
Sbjct  61   ........................................CG....A...........A.  120
Query  121  AGCCGTTATGGTACAAACTTGAATGGCTTGCTCAATCTTTTGTCCACCAAAGTTCCTTCA  180
Sbjct  121  ....C.......T...................T..A..A.....................  180
Query  181  AAAGGGTTTGGTCTCAGCTCGACTGGGCAAGGCCAAGATCGAGCAAATGGTTTAGCCCTA  240
Sbjct  181  ....................A................C..A...................  240
Query  241  TGCCGGGGTGATGTCTCAAAAACAAACTGTACGACCTGTGTCATTGATGCAGGCAAAGAG  300
Sbjct  241  .....A.........................A............................  300
Query  301  CTTGGTAATCGTTGTCCTTATAAAAAAGGAGCGATAATTTGGTATGATAACTGTCTTTTG  360
Sbjct  301  ...........................................................A  360
Query  361  AAGTACTCGAACATTGATTTCTTTGGAGAAATCGATAACAAAAACAAGTTCTACATGTGG  420
Sbjct  361  ..............A.............................T...............  420
Query  421  AACGTCCAAGATGTAGAAAATCCCACTTCATTCAATCCAAAAGTTAAGGATTTGTTAAGC  480
Sbjct  421  ......G.....................................................  480
Query  481  AGGTTATCTAATAAAGCTTATGCCAATCCAAAATTCTATGCTACCGGGGACCTAAAGCTT  540
Sbjct  481  ............................................................  540
Query  541  GACTCATCAAGCAAACTATATGGTTTGGCTCAATGCACCAGGGACCTATCAGGTCTTGAT  600
Sbjct  541  ..T.................C..........................T............  600
Query  601  TGTAAGAAGTGTCTTGATACTGCGATTAGTGAACTTCCCAACTGTTGCGATGGAAAACGA  660
Sbjct  601  ............................................................  660
Query  661  GGTGGGCGAGTTGTTGGTGGCAGTTGTAACGTTAGATATGAACTTTACCCCTTTGTTGAT  720
Sbjct  661  ............................................................  720
Query  721  GCCTAG  726
Sbjct  721  ......  726
```

The points indicate equal nucleotides.

Program: **blastp** suite-2sequences (NCBI)
Query: Cast_Gnk2-like (amino acid) Length: 241
Subject: Qs_Gnk2-like (amino acid) Length: 241. NCBI ref. XM_024067951.1

Alignment:
Score:417 bits(1072), Expect:1e-154,
Method:Compositional matrix adjust.,
Identities:228/241(95%), Positives:233/241(96%), Gaps:0/241(0%)

```
Query  1    MLSSKYISVSFLLLSLSLHAVNCADPLYHFCFSQESYTATSRYGTNLNGLLNLLSTKVPS  60
Sbjct  1    ..C...F...........Y..............P.N...N.P..S......K........  60

Query  61   KGFGLSSTGQGQDRANGLALCRGDVSKTNCTTCVIDAGKELGNRCPYKKGAIIWYDNCLL  120
Sbjct  61   ............AQ................K.............................  120

Query  121  KYSNIDFFGEIDNKNKFYMWNVQDVENPTSFNPKVKDLLSRLSNKAYANPKFYATGDLKL  180
Sbjct  121  ......................E.....................................  180

Query  181  DSSSKLYGLAQCTRDLSGLDCKKCLDTAISELPNCCDGKRGGRVVGGSCNVRYELYPFVD  240
Sbjct  181  ............................................................  240

Query  241  A  241
Sbjct  241  .  241
```

The points indicate equal amino acids. The amino acids that are predicted to be part of the signal peptide for secretion (SignalP 6.0 - https://services.healthtech.dtu.dk/services/SignalP-6.0/) are in blue. The amino acids in red, highlighted by red arrows, indicate the cysteines part of the DUF26 conserved domains (C-X8-C-X2-C) potentially involved in the host defense to the pathogen.

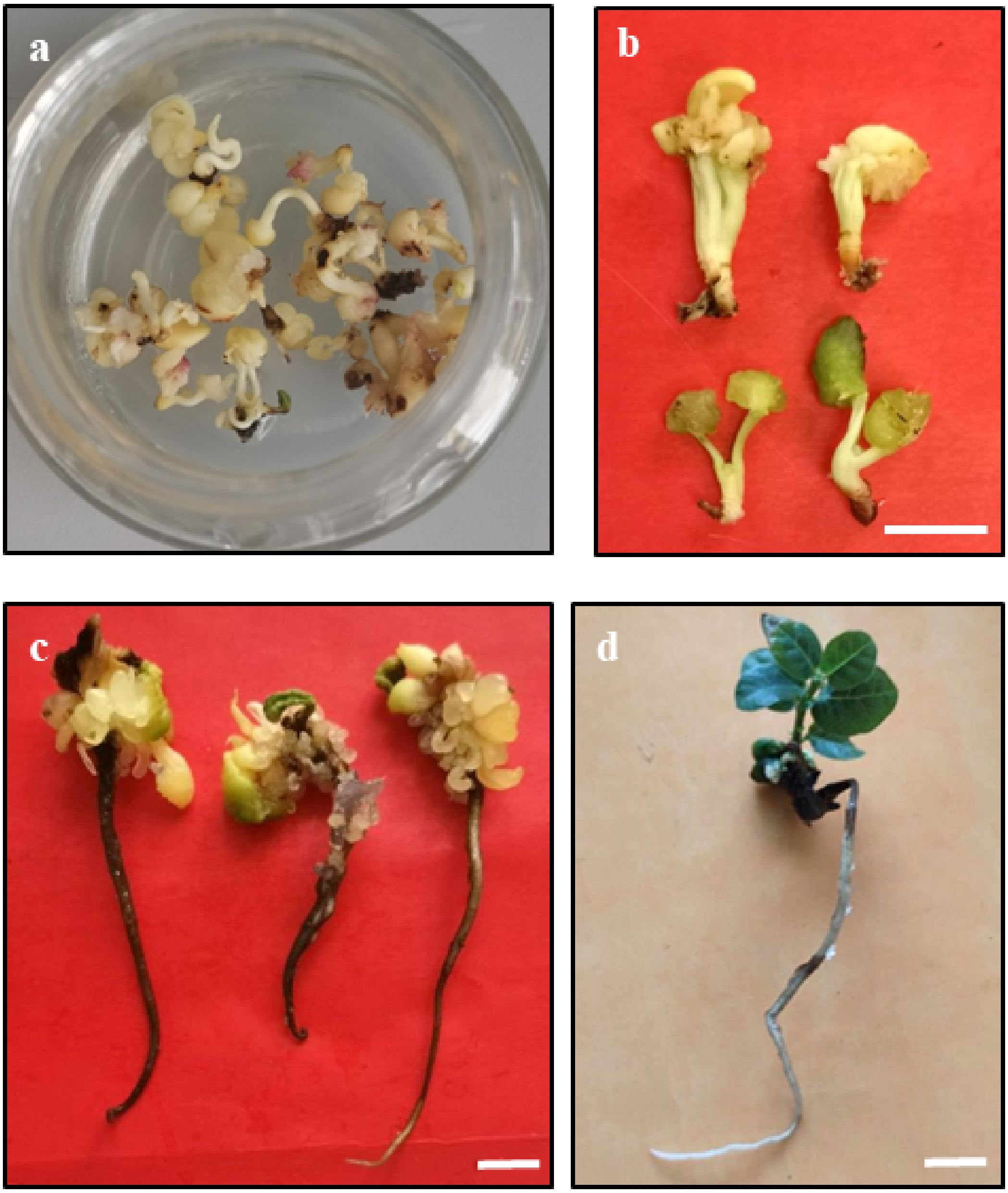

Supplementary Information 3. Different steps and responses during the plant regeneration of cork oak somatic embryos overexpressing *Cast_Gnk2_like* gene. a, b Morphological aspect of somatic embryos after 2 months of cold storage. c Transgenic somatic embryos (left and right) and non-transgenic somatic embryos (center) showing only root development following two months of cold storage and eight weeks on germination medium. d Transgenic plantlet following to two months of cold storage and eight weeks on germination medium. a Diameter of the glass jar, 55 mm. b, c, d Bar, 1 cm.

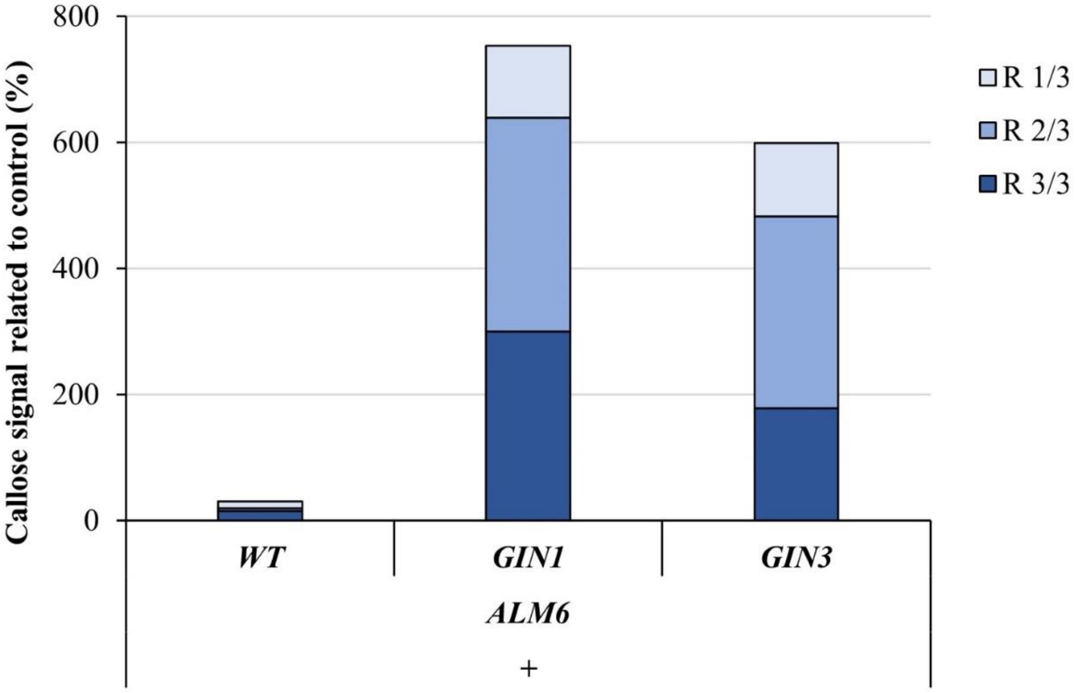

Supplementary Information 4. Quantification of callose deposition in *Quercus suber* roots. Callose deposition was quantified with ImageJ® program. The digital photos were acquired at 100x magnification. Bar: (1 mm). Staining with Aniline Blue to detect callose deposition on the roots of wild type (WT), and the transgenics ALM6-GIN 1 and ALM6-GIN 3, inoculated with *Pc* zoospores (10^7 zoospores/ml), and related, in percent, to non-inoculated control signal (water), after four days after inoculation. Data and photos were taken from at least tree explants taking at least five photos from three different root areas (1. Differentiation, 2. elongation, and 3. meristematic). Quantification corresponds to photos of Figure 5d.

Supplementary Information 5. Culture media used in the different steps of genetic transformation and plant regeneration using somatic embryos of cork oak as target explants.

Components	Proliferation medium	Preculture/Coculture medium	Infection medium	Selection medium	Germination medium
**Basal medium**	SH	MS	MS	SH	SH
**BA (mg/l)**	-	-	-	-	0.025
**IBA (mg/l)**	-	-	-	-	0.05
**Sucrose (g/l)**	30	30	50	30	30
**Plant Propagation agar (g/l)**	6	6	-	6	6
**Kanamycin (mg/l)**	-	-	-	125	-
**Carbenicilline (mg/l)**	-	-	-	300	-
**pH**	5.6-5.7	5.6-5.7	5.6-5.7	5.6-5.7	5.6-5.7

BA, 6-benzyladenine; IBA, indole-3-butyric acid; MS, Murashige and Skoog (1962); SH, Schenk and Hildebrandt (1972).

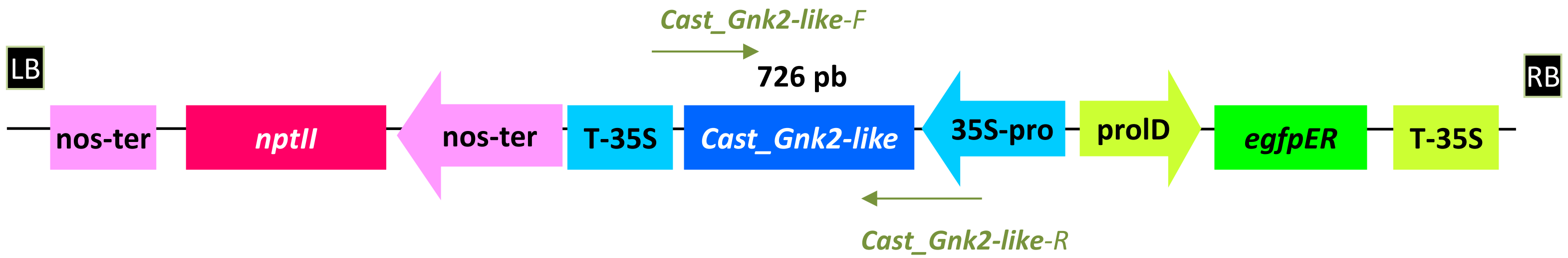

Supplementary Information 6. A graphic diagram of T-DNA region of the vector pK7WG2D-GIN. *nptII* neomycin phosphotransferase marker gene; nos-ter, nos-pro terminator and promoter of nopaline synthase gene, respectively; *Cast_Gnk2-like* gene encoding a Ginkbilobin2-like protein; 35S-pro and T-35S promoter and terminator of Cauliflower mosaic virus gene, respectively; *egfpER* green fluorescence protein gene; prolD rol root loci D promoter; RB right border; LB left border.
